# Comparison of patellofemoral contact pressure after semi-cylindrical recession trochleoplasty and trochlear block recession in feline cadavers

**DOI:** 10.3389/fvets.2023.1237291

**Published:** 2023-08-23

**Authors:** Goda Choi, Jinsu Kang, Namsoo Kim, Suyoung Heo

**Affiliations:** Department of Surgery, College of Veterinary Medicine, Jeonbuk National University, Iksan-si, Republic of Korea

**Keywords:** patellar luxation, trochlear hypoplasia, trochleoplasty, patellofemoral joint contact mechanism, feline

## Abstract

**Introduction:**

The purpose of this study was to compare the changes in the patellofemoral joint (PFJ) contact mechanisms of the normal state, trochlear hypoplasia model and after performing trochleoplasty on the hypoplasia model in feline cadavers.

**Methods:**

Twenty normal pelvic limbs were acquired from the 10 feline cadavers. First, the PFJ contact mechanisms were measured in normal state, then trochlear hypoplasia models were created using customized trochlear ridge cutting guides. After measuring PFJ contact mechanisms in the trochlear hypoplasia models, they were divided into two groups and performed semi-cylindrical recession trochleoplasty (SCRT) and trochlear block recession (TBR) were performed, respectively. After that, the PFJ contact mechanisms were measured and the values of the 4 groups (normal state, trochlear hypoplasia, SCRT, TBR) were compared.

**Results:**

The trochlear hypoplasia group showed increased contact pressure and decreased contact areas compared to the normal state group. In the groups that underwent tracheoplasty (SCRT and TBR), PFJ contact mechanisms were recovered similarly to that of the normal state group. The PFJ of the SCRP group was measured similar to that of the normal group than that of the TBR group.

**Discussion:**

Tracheoplasty can be useful in restoring PFJ contact mechanisms and SCRT can be considered as a good alternative to the conventional methods of trochleoplasty.

## 1. Introduction

Patellar luxation (PL) is rarely reported in cats compared to dogs, but is quite common in the Devon Rex and Abyssinian breeds (1, 2). A previous study reported that medial, bilateral, and developmental PL are more common in cats than lateral, unilateral, and traumatic PL (1). Developmental feline PL is associated with trochlear hypoplasia (TH), under-developed medial femoral condyle, and medial or lateral deviation of the tibial tuberosity (3). The patellar stabilization is more challenging for cats than for dogs because cats’ patella is loosely positioned and broader relative to the trochlea (2). Surgical options for PL include trochleoplasty, tibial tuberosity transposition, imbrication and/or release of soft tissue, and femoral corrective osteotomies (4).

Trochleoplasty corrects patellofemoral joint (PFJ) with inadequate trochlear depths (5). Techniques to deepen femoral trochlea include trochlear chondroplasty, trochlear wedge recession (TWR), and trochlear block recession (TBR) (5). TBR and TWR are techniques that can recess the femoral trochlea while preserving most of the articular cartilage (5, 6). Surgeons prefer techniques that preserve articular cartilage, as it results in slower developments of osteoarthritis and faster recoveries (5, 7–9). Recently, semi-cylindrical recession trochleoplasty (SCRT) has been used in dogs and no significant difference in clinical outcome was found compared to TBR. In previous report, TBR and SCRT show no significant difference in clinical outcomes (10).

In human medicine, trochlear dysplasia (TD) is the cause of patellar subluxation (11). Human patients with TD have shown an increase in contact pressure of the patellofemoral joint (PFJ) and a decrease in contact area (12). These changes in contact mechanisms can influence the development of various joint diseases (13–15). When patients with TD undergo trochleoplasty, it has been observed that their increased contact pressure decreases, and their decreased contact area increases (16).

To the best of the author’s knowledge, there is no study in veterinary medicine on changes in PFJ contact mechanisms (contact pressure and contact area) with TH and changes after trochleoplasty. This study compares the changes in the contact mechanisms in the normal PFJ and in the hypoplasia PFJ, and the changes after trochleoplasty (SCRT and TBR) in the PFJ with TH. Our first hypothesis is that the PFJ with TH would have the increased contact pressure and the decreased contact area compared to the normal PFJ. The second hypothesis is that there would be decreased contact pressure and increased contact area after trochleoplasty.

## 2. Materials and methods

### 2.1. Animals

This study was approved by the Institutional Animal Care and Use Committee of Jeonbuk National University (JBNU 2022-035). Twenty cadaveric hindlimbs were obtained from 10 adult cats, euthanized for reasons unrelated to this study. The cadavers were selected based on the criteria of not having any visually apparent orthopedic conditions. Computed tomography (Alexion TSX-034A, Toshiba, Tokyo, Japan) examinations revealed no stifle joint lesions. The specimens were bilaterally disarticulated at the coxofemoral joint from each cadaver. All soft tissues of the specimens were dissected from the distal calcaneus to the proximal femur, except for the stifle, hook joint capsule, and collateral ligaments. The quadriceps muscle tendon was preserved approximately 1.5 cm from its proximal attachment. Each specimen was stored in 0.9% normal saline, wrapped in a plastic bag, and frozen at −80°C.

**Figure 1:**
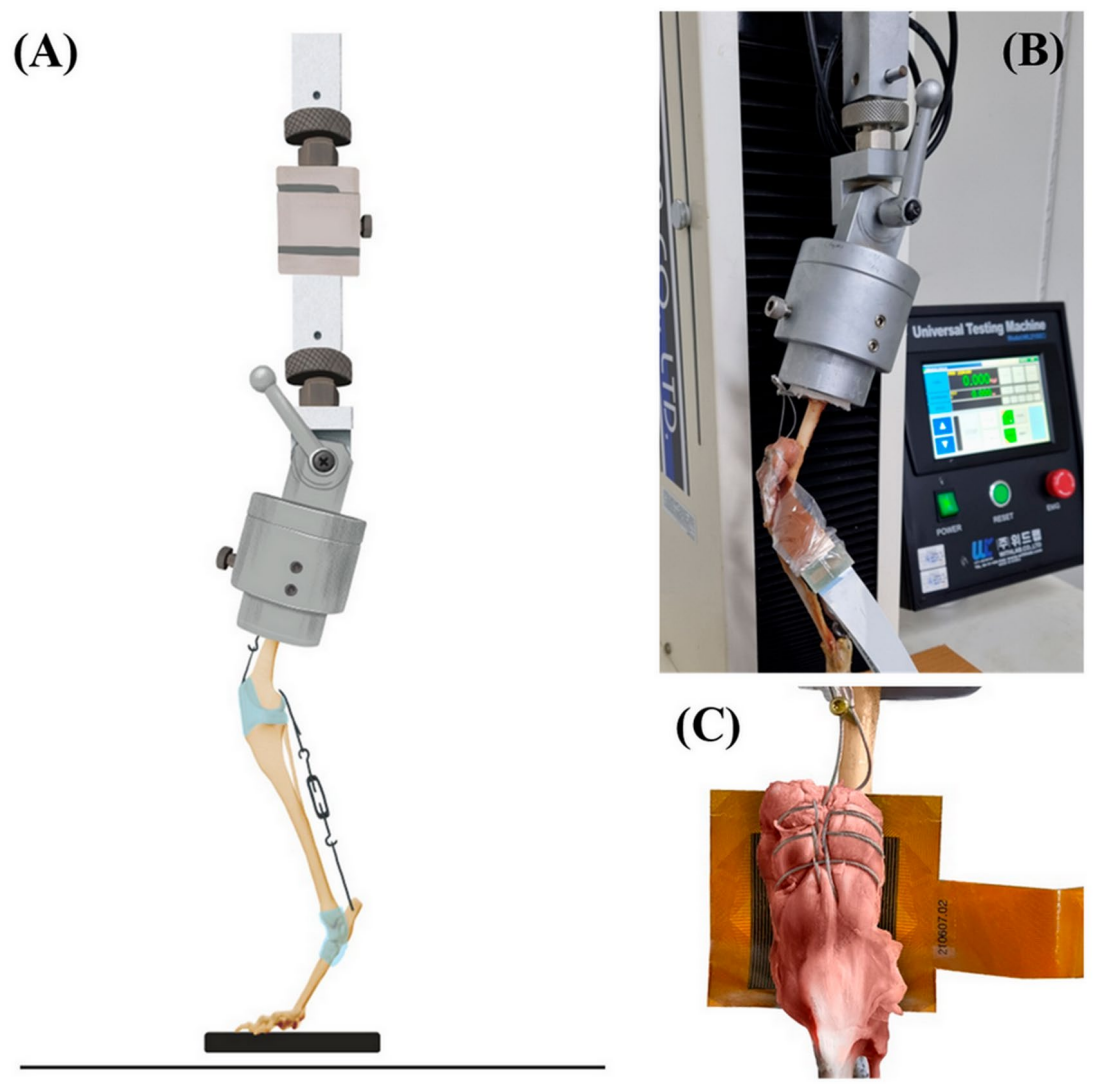
The apparatus of PFJ contact mechanisms. The illustration of specimen within the custom testing jig attached to the mechanical testing machine **(A)**. The stifle and hook joint angles were both adjusted to 120° ± 5° with the turnbuckles. The force-sensitive resistor placed between the patella and the trochlear groove **(B)**. Magnified illustration of the resistor insertion **(C)**.

In preparation for the experiment, the specimens were thawed at room temperature under moisture gauzes. The setting of the experiment was modified from selected previous studies (17, 18). A Krackow suture was performed on the quadriceps muscle using a braided steel cable, and the cable was secured by connecting it to a screw placed at the proximal femur, imitating the mechanism of the quadriceps muscle. Another braided steel cable was passed between the bilateral fabella and the femoral condyle. After a hole was drilled at the proximal mid-point of the calcaneus using a 1.5-size Kirschner wire, another braided steel cable was passed through the hole. The two cables were installed to mimic the hamstring mechanism (Figure 1A).

### 2.2. Experimental design

In normal state group, the femoral head and greater trochanter of the specimens were held in a customized jig by a commercially available acrylic resin, in a normal feline stand phase. The angle between the femoral diaphysis and the top side of the jig was set to 70° (19, 20). The jig was installed on a Universal Testing Machine (WL2100C, Withlab Inc., Gunpo, South Korea). Stifle and talocrural angles were both adjusted to 120°± 5° with the turnbuckles (17). The angles were measured by an electronic goniometer. The pressure on the PFJ was measured as a load of 30% body weight in the specimens (18). To prevent slipping, commercially available sandpaper was attached to the contact area of the palmar pad, with the force-sensitive resistor (TFM 4800, Kitronyx Inc., Seoul, South Korea) placed between the patella and the trochlear groove (Figures 1B,C). The force-sensitive resistor was 7.2 cm × 7.2 cm (0.12 mm thickness) piezoresistive-type and equipped with a total of 2,304 nods. To fix the position and height of the resistor, the force controller (Bikal L; Kitronyx Inc.) connected to the force-sensitive resistor was secured using a height and angle adjustable tablet holder.

**Figure 2:**
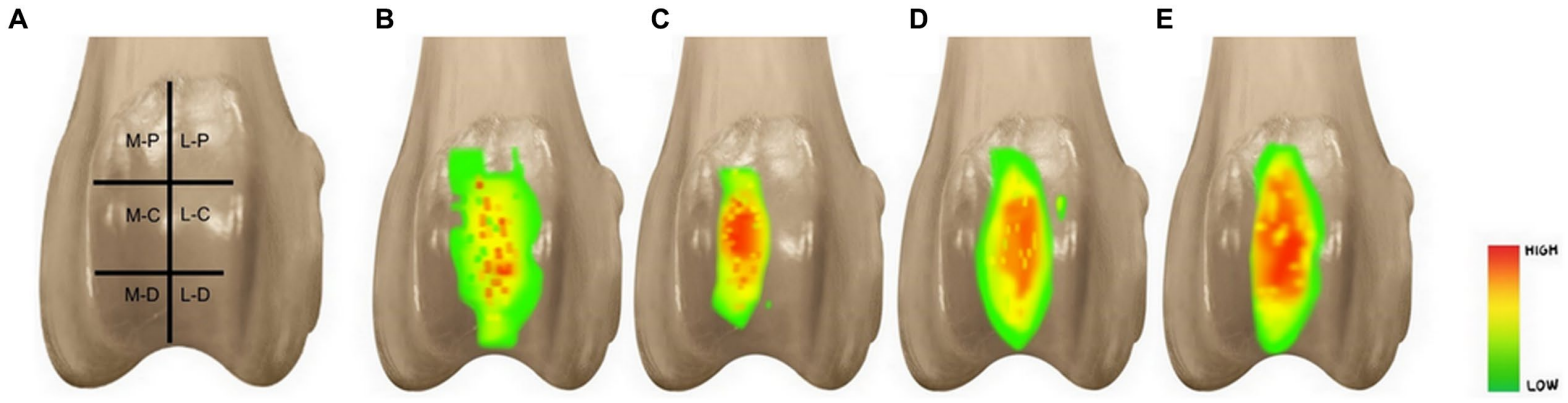
Illustration of six areas on the trochlear groove **(A)**. According to the anatomical location of the trochlear groove was divided in six areas. And they were named medial-proximal area (M-P), lateral-proximal area (L-P), medial-center area (M-C), lateral-center area (L-C), medial-distal area (M-D), and lateral-distal area (L-D). Visualization of the pressure distribution of patellofemoral joint of each four groups. Visualization of the pressure distribution of patellofemoral joint of each four states **(B–D)**. Normal state **(B)**. Trochlear hypoplasia **(C)**. Semi-cylindrical recession trochleoplasty **(D)**. Trochlear block recession **(E)**.

The contact pressure and contact area were measured in the following six areas: medial-proximal (M-P), lateral-proximal (L-P), medial-center (M-C), lateral-center (L-C), medial-distal (M-D), and lateral-distal (L-D). Each experiment lasted for 5 s under a load of 30% body weight and was repeated five times. The results are the mean of these 5 measurements. For M-P, L-P, M-D, and L-D areas, the range of 72 (6 × 12) cells was set, respectively, and for M-C and L-C areas, the range of 144 (12 × 12) cells was set (Figure 2A).

**Figure 3:**
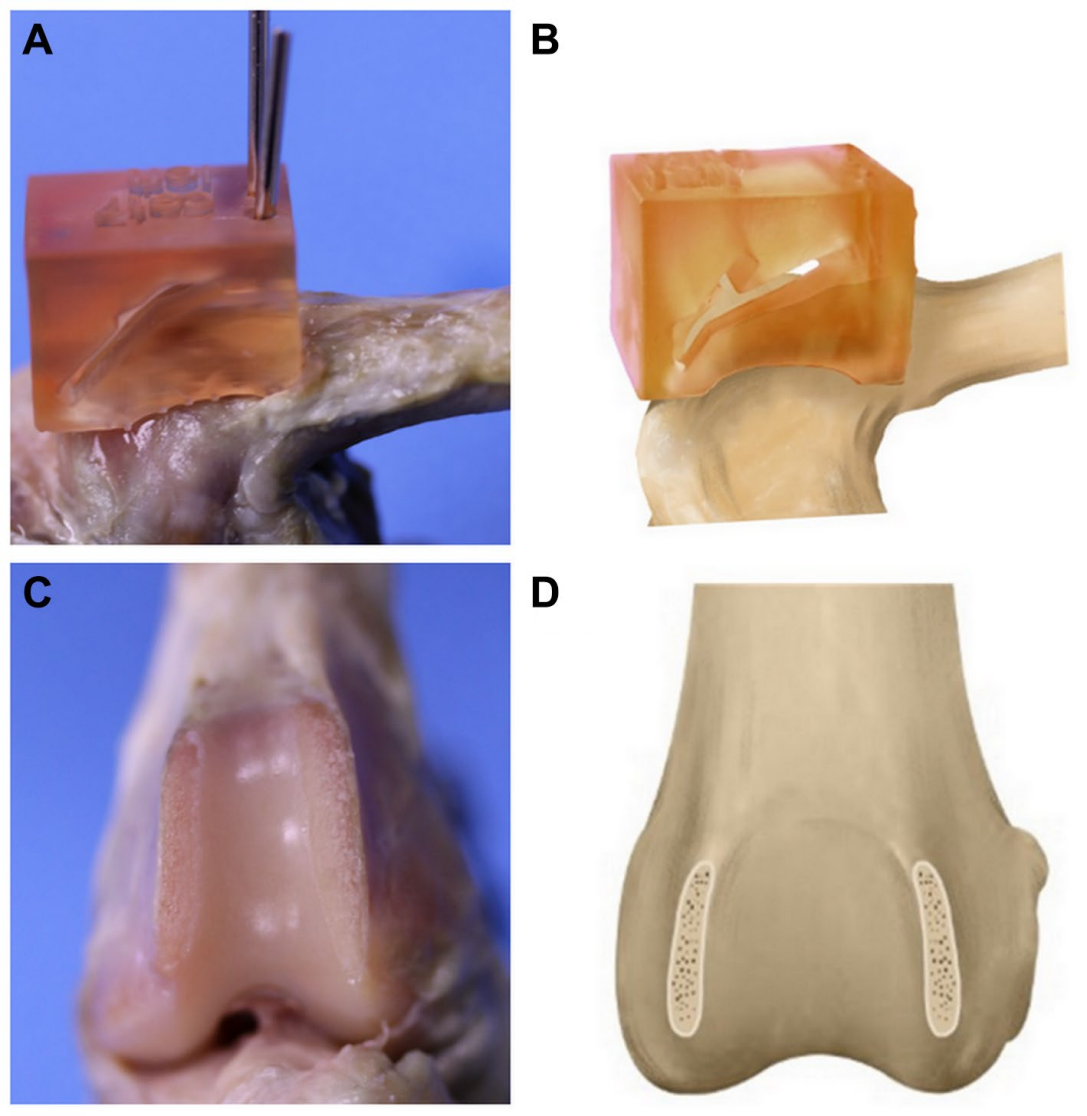
Production of a trochlear hypoplasia (TH) model. The specimen-customized cutting guide is installed on the bone **(A)**. The illustration of guide installation **(B)**. After the cutting a hyaline cartilage and subchondral bone from the trochlear ridge **(C)**. The illustration of TH model **(D)**.

**Figure 4:**
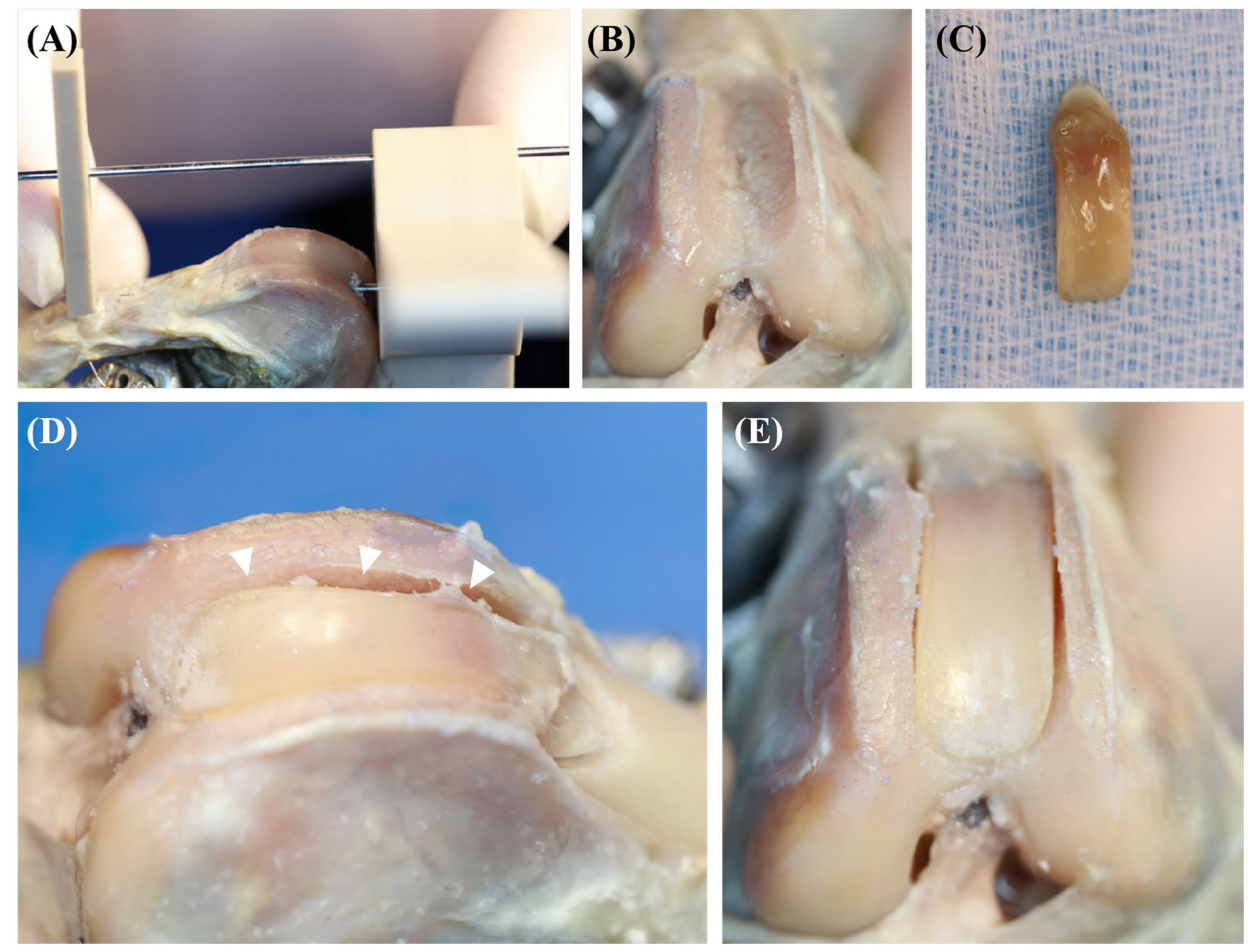
The process of semi-cylindrical recession trochleoplasty (SCRT) **(A–E)**. White arrowheads in **(D)** indicates the depth deepened by SCRT.

To mimic clinical situations, TH models were created in all hindlimbs. The specimen-customized cutting guide was designed so that the height of both trochlear ridges is 5 mm from the trochlear groove and removed hyaline cartilage and subchondral bone from the trochlear ridge (Figure 3). The PFJ contact mechanisms were measured in the same methods as in the normal state. TBR and SCRT were performed on each specimen. The limbs were randomly divided between the two groups using randomization software.^1^ TBR was performed on one hindlimb and SCRT was performed on the opposite hindlimb (Figure 4), and the surgical method referred to the existing literature (10, 19). The PFJ contact mechanisms were measured in the same methods as in the normal state.

The resistor data were converted using the manufacturer’s software (PetLAB, Kytronix Inc.). In this experiment, the active sensing area was divided by the following criteria. The top 10% of the recorded total numerical values were highlighted in color, and the procedures were performed following the mentioned order in comparison with the visual pressure map. In the sensing area, the contact pressure represents the sum of all measured numbers, and the contact area represents the sum of the number of cells which numbers were measured.

### 2.3. Statistical analysis

Continuous data were evaluated for a normal distribution using the Kolmogorov–Smirnov test. All were found to meet the assumptions of a normal distribution and were reported as mean and standard deviation values. The Kruskal-Wallis test was used to compare values among normal state group (NSG), trochlear hypoplasia model group (THG), SCRT group (SCRTG), and TBR group (TBRG). The Post-hoc Mann–Whitney test was used to compare values of each pairwise, respectively. Statistical significance was set at *p* < 0.001. Statistical analyses were performed using SPSS version 26.0 (IBM, Armonk, NY, United States).

## 3. Results

### 3.1. Cadaveric specimens

The body weight of the cadavers was from 1.9–5.2 kg.

### 3.2. Data visualization

The contact area was measured by using Desktop Ruler version 3.8.6498 (AVPSoft, Moscow, Russia), which allowed us to ascertain the width in the horizontal and vertical dimensions. It was visually confirmed that the pressure distribution of the THG had a narrower contact area and a was biased medially compared to NSG (Figures 2B,C). In addition, it was visually emphasized and confirmed that the contact pressure had a high intensity in THG (Figure 2C). It was visually estimated that the contact area and contact pressure became close to the normal state in SCRTG and TBRG (Figures 2D,E).

### 3.3. The contact pressures

**Figure 5:**
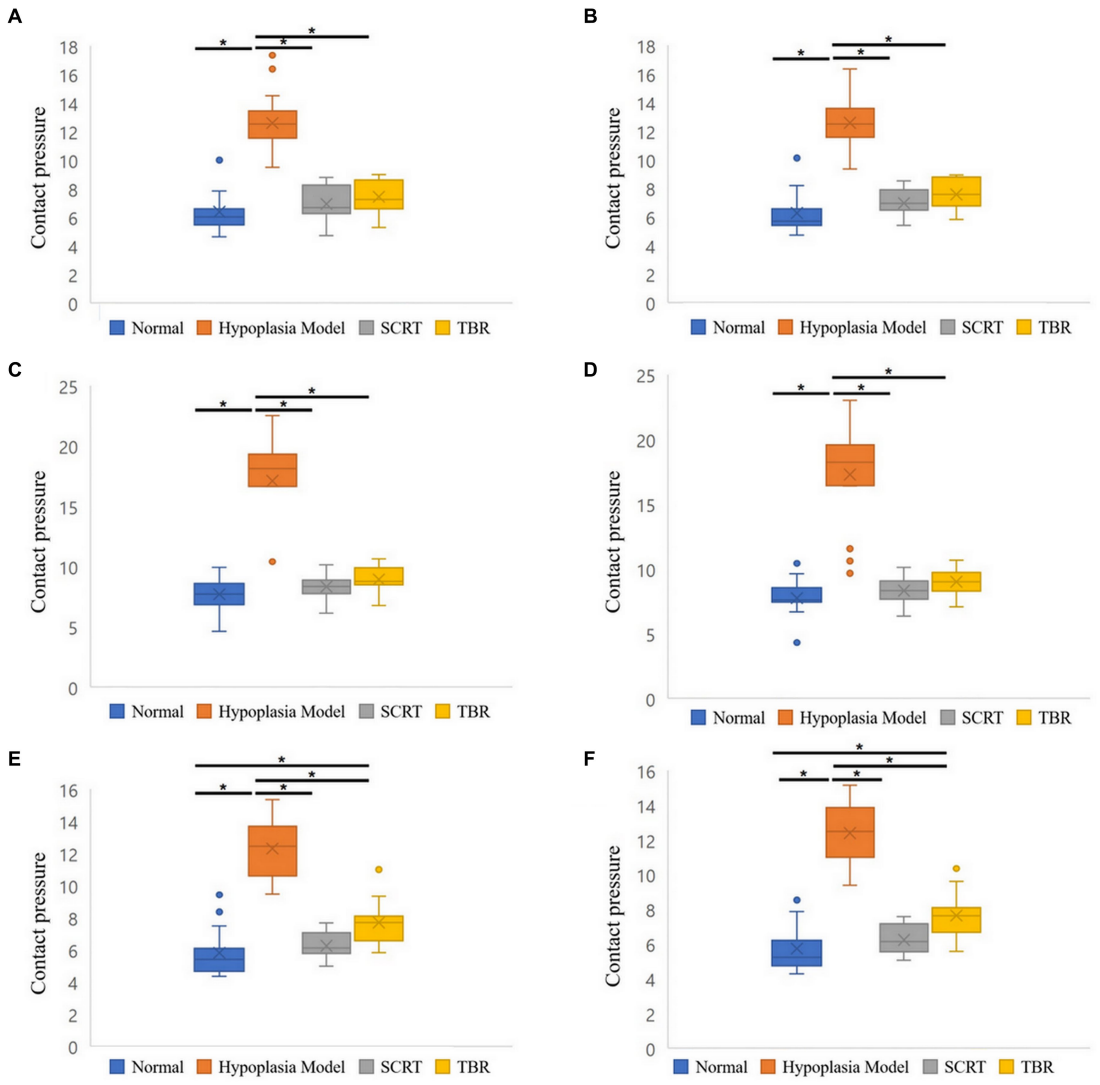
Boxplots of the contact pressure of six areas of patellofemoral joint. Medial-proximal (M-P) area **(A)**. Lateral-proximal (L-P) area **(B)**. Medial-center (M-C) area **(C)**. Lateral-center (L-C) area **(D)**. Medial-distal (M-D) area **(E)**. Lateral-distal (L-D) area **(F)**. Significant differences between each four groups: ^*^p < 0.001.

The contact pressure of the THG was increased than that of NSG (*p* < 0.001). Specifically, it increased by 1.98–2.22 times in all areas. The contact pressure was decreased in two groups (SCRTG and TBRG) compared to the THG. First, in the SCRTG, compared to the THG, the contact pressure decreased by 45%–52% in all areas. Then, the contact pressure reduction rates of the TBRG and the THG were as follows: 41% decrease in the M-D area, 40% decrease in the L-D area, 56% decrease in the M-C area, 48% decrease in the L-C area, 37% decrease in the M-D area, and 38% decrease in the L-D area. Comparing the NSG and the SCRTG, the SCRTG showed a higher contact pressure in all areas as follows: 9% in the M-P area, 11% in the L-P area, 8% in the M-C area, 7% in the L-C area, 7% in the M-D area, and 9% in the L-D area. When contact pressures were compared between the TBRG and the NSG, the results were different from the case of the SCRTG and the NSG. First, in the M-P and L-P areas, the contact pressure of the TBRG was higher than the NSG. In the M-C area, the contact pressure of the TBRG 2% is lower than NSG, and in the L-C area, it is 16% higher than NSG. In the M-D and L-D areas, the contact pressure of the TBRG were 33% higher than that of the NSG (Figure 5).

### 3.4. The contact area

**Figure 6:**
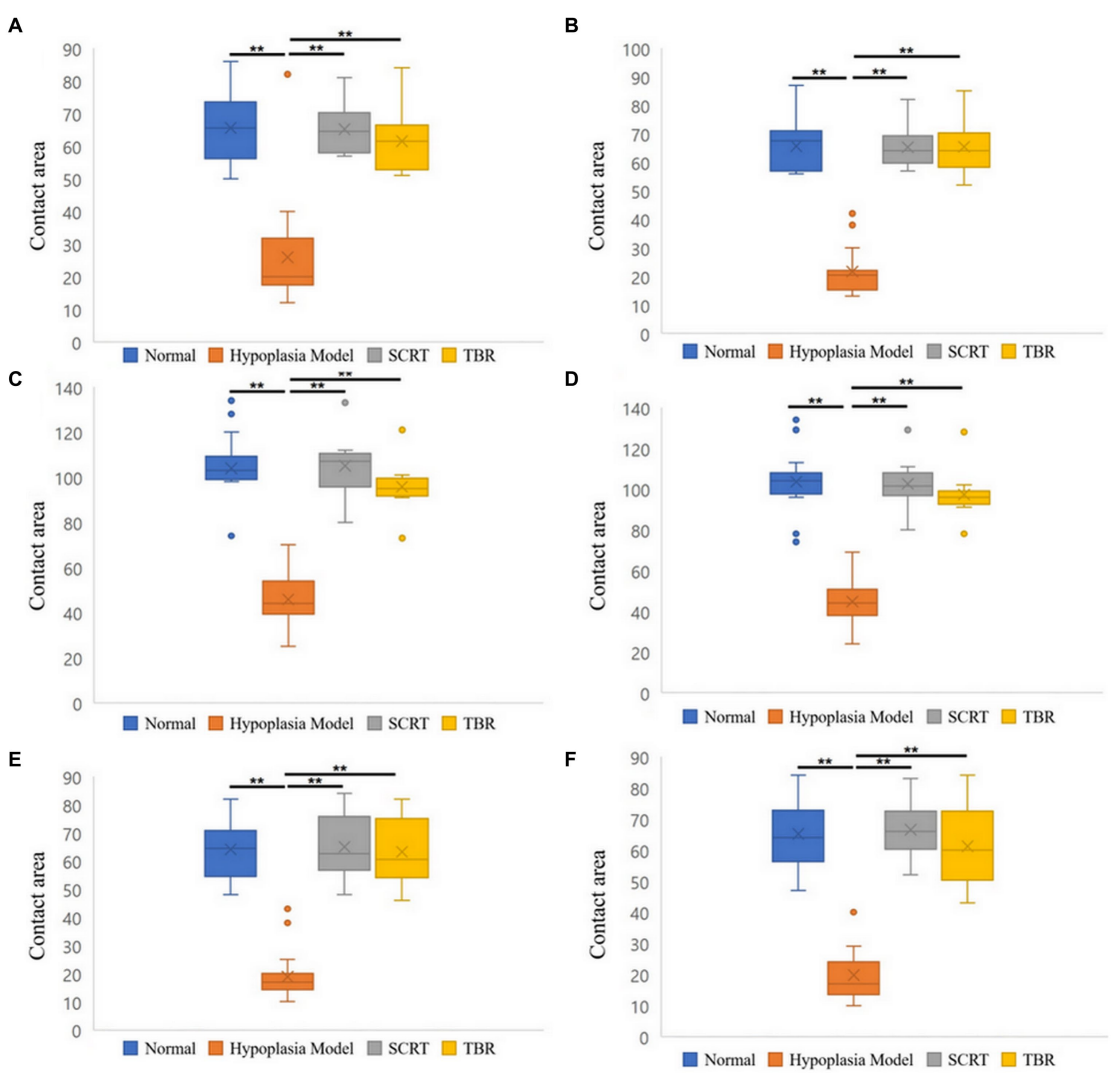
Boxplots of the contact area of six areas of patellofemoral joint. Medial-proximal (M-P) area **(A)**. Lateral-proximal (L-P) area **(B)**. Medial-center (M-C) area **(C)**. Lateral-center (L-C) area **(D)**. Medial-distal (M-D) area **(E)**. Lateral-distal (L-D) area **(F)**. Significant differences between each four groups: ^**^p < 0.001.

When the contact area between the NSG and the THG was compared, it decreased in the THG than in the NSG. In the M-P area, it was reduced by 60%; in the L-P area, by 67%; in the M-C area, by 56%; in the L-C area, by 57%; in the M-D area, by 71%, and in the L-D area, by 70% (for all, *p* < 0.001). The difference in the mean contact area between the SCRTG and the NSG was found to be 1%. In the M-P, L-P, and L-C areas, the average contact area of the SCRTG was 1% lower than that of the NSG, while the M-C, M-D, and L-D areas showed an increase of about 1%. Comparing the contact areas between the TBRG and the NSG, it was found that the contact area of the TBRG decreased in most area. In the L-P area, there was no difference between the TBRG and the NSG. However, in the remaining 5 areas (M-P, M-C, L-C, M-D, and L-D), the contact area of the TBRG decreased compared with the case of the NSG: 6% in the M-P area, 8% in the M-C area, 6% in the L-C area, 1% in the M-D area, and 6% for the L-D area (Figure 6).

## 4. Discussion

This study compared the PFJ contact mechanism (contact pressure and contact area) of the normal state, the trochlear hypoplasia, and the trochleoplasty (SCRT and TBR) groups. Consistent with our first hypothesis, in this study, it was found that the contact pressure was increased and the contact area was decreased in the trochlear hypoplasia compared to the normal state. But the second hypothesis that there would be no difference in contact pressure between the trochleoplasty (SCRT and TBR) and the normal state is rejected.

In this study, in the THG, the mean contact pressure was increased and the contact area was decreased than the NSG. The contact pressure of the SCRTG increased in all 6 areas compared to the NSG. Additionally, the contact pressure of the TBRG decreased by 2% compared to the NSG in the M-C area while it increased in the other five areas. This confirmed that the contact pressure was reduced through trochleoplasty but remained higher than normal. The contact pressure in the distal area was 33% higher in the TBRG than in the NSG. In TBR procedures, distal trans-trochlear cuts are made perpendicular to the trochlear sulcus and a straight basilar cut connects proximal and distal trans-trochlear margins (19). Realistically, the side-effects of osteotomy procedures include higher contact pressures (18).

Human TD patients showed increased contact pressure and decreased contact area (12), which is consistent with the results of this experiment. Overloading the cartilage is of particular concern as a possible source of pain and articular cartilage degeneration (21). Most patients with severe patella luxation have degenerated trochlear cartilage and complain of pain. In dogs with medial patellar luxation, excessive pressure loaded on the trochlear causes pain and degeneration of articular cartilage (7). Previous experimental studies in human medicine have demonstrated that contact pressure and contact area strongly contribute to the development of osteoarthritis (13–15). Additionally, in previous report identified changes in the patellar lateral facets, with shearing at the highest pressure, in an *in vivo* induced-lateral patellar subluxation rabbit model (22).

In the contact area, the difference between the SCRTG and the NSG was 1% in all 6 areas. On the other hand, in the TBRG, there was no difference between the L-P area and the NSG, but the contact area decreased in the remaining 5 areas. This study appears to show that SCRT restore the contact area closer to the normal than TBR. The visualization of pressure distribution also confirms that, although statistical comparison is difficult, SCRTG is more like the NSG than TBRG.

In humans with patellar subluxation, the patellofemoral cartilage contact areas were significantly smaller than in a healthy state. This likely involves an increased contact pressure in patients, as a similar force is transmitted through a much smaller surface (23). Therefore, it is presumed that the contact pressure in the THG was increased compared to the NSG due to the above-mentioned reasons. Comparing the THG and both trochleoplasty groups (SCRTG and TBRG), the mean contact pressure decreased and the contact area increased in both trochleoplasty groups, which is consistent with deepening trochleoplasty-treated TD patients in human side (16).

This study has some limitations. First, the cadaveric feline stifle model was modified from a previous *ex vivo* model (18). Although it reproduced the quadriceps and hamstring muscle mechanism in a standing posture for feline stifles *in vivo*, it cannot represent all elements. Moreover, dissections of additional joint capsules and soft tissues were necessary to insert the force-sensitive resistor and perform the trochleoplasty procedures in the stifle joint. In addition, the model could not simulate dynamic biomechanical environments during activity *in vivo* (24). Second, it is difficulty in fixing the force-sensitive resistor to the femoral trochlear and avoiding crinkling of the resistor because of the slippery and irregular articular surface. These problems may have influenced the validity of the data and caused artifacts. Third, maintaining the angles of the stifle and talocrural 120° ± 5°, respectively, was difficult, and adjustments of the flexion angle resulted in unstable apparatus, as in previous studies (17). For this, the evaluation of diverse flexion angles was restricted in this study. Fourth, the small sample size is small and breeds of the cats were biased. This made it difficult to assess the difference in pressure distribution among different breeds.

## 5. Conclusion

In conclusion, TH increases the contact pressure and decreases the contact area, trochleoplasty can help normalize PFJ mechanism. When comparing SCRT and TBR, it suggests that SCRT may tend to restore close to the normal PFJ mechanism. In addition, SCRT can be considered as a good alternative to the conventional trochleoplasty methods.

## Data availability statement

The original contributions presented in the study are included in the article/supplementary material, further inquiries can be directed to the corresponding author.

## Ethics statement

The animal study was reviewed and approved by Jeonbuk National University Institutional Animal Care and Use Committee (IACUC).

## Author contributions

GC and SH: conception, design, and draft of the manuscript. GC, JK, and SH: production of the specimen-customized cutting guide. GC: performing surgical procedures. GC, JK, NK, and SH: data analysis and interpretation. NK and SH: revising the article for intellectual content and final approval of the completed article. All authors contributed to the article and approved the submitted version.

## Conflict of interest

The authors declare that the research was conducted in the absence of any commercial or financial relationships that could be construed as a potential conflict of interest.

## Publisher’s note

All claims expressed in this article are solely those of the authors and do not necessarily represent those of their affiliated organizations, or those of the publisher, the editors and the reviewers. Any product that may be evaluated in this article, or claim that may be made by its manufacturer, is not guaranteed or endorsed by the publisher.

## Footnotes

^1^ http://www.random.org/

## Abbreviations

PFJ: Patellofemoral joint
SCRT: Semi-cylindrical recession trochleoplasty
TBR: Trochlear block recession
PL: Patellar luxation
TH: Trochlear hypoplasia
TWR: Trochlear wedge recession
TD: Trochlear dysplasia
NSG: Normal state group
THG: Trochlear hypoplasia model group
SCRTG: Semi-cylindrical recession trochleoplasty group
TBRG: Trochlear block recession group
